# Differential Expression and Alternative Splicing of Transcripts Associated With Cisplatin-Induced Chemoresistance in Nasopharyngeal Carcinoma

**DOI:** 10.3389/fgene.2020.00052

**Published:** 2020-02-25

**Authors:** Jian Zhang, Huali Jiang, Tao Xie, Jieling Zheng, Yunhong Tian, Rong Li, Baiyao Wang, Jie Lin, Anan Xu, Xiaoting Huang, Yawei Yuan

**Affiliations:** ^1^ Department of Radiation Oncology, Affiliated Cancer Hospital & Institute of Guangzhou Medical University, State Key Laboratory of Respiratory Diseases, Guangzhou Institute of Respiratory Disease, Guangzhou, China; ^2^ Department of Cardiovascularology, the Affiliated Donghua Hospital of Sun Yat-sen University, Dongguan, China; ^3^ Department of Radiation Oncology, Nanfang Hospital, Southern Medical University, Guangzhou, China

**Keywords:** transcriptome sequencing, cisplatin, chemoresistance, nasopharyngeal carcinoma, differentially expressed gene, alternative splicing

## Abstract

Radiotherapy and adjuvant cisplatin (DDP) chemotherapy are standard administrations applied to treat nasopharyngeal carcinoma (NPC). However, the molecular changes and functions of DDP in NPC chemo-resistance remain poorly understood. In the present study, transcriptomic sequencing between 5-8F and 5-8F/DDP cells was performed to identify differential expression and alternative splicing (AS) characteristics in DDP-resistant NPC cells. Transcriptomic profiling identified 1,757 upregulated genes and 1,473 downregulated differentially expressed genes (DEGs). Bioinformatic analysis revealed that these DEGs were associated with or participated in important biological regulatory functions in NPC. Validation of 20 signiﬁcant DEGs using quantitative real-time reverse transcription PCR showed that the expression patterns of 17 mRNAs were in accordance with the sequencing data. Intron retention was identified as the major AS event in chemoresistant cells. Furthermore, the expression level of matrix metalloproteinase 1 (*MMP1*), which was one of the most upregulated mRNAs in the chemoresistant cell lines, was signiﬁcantly associated with the migration, invasion, and proliferation of NPC cells *in vitro*. Our study revealed that dysregulated genes and AS-mediated DDP chemoresistance might play important roles in NPC development and progression. Targeting aberrantly expressed genes might clarify the pathogenesis of NPC and contribute to developing new therapeutic strategies for NPC.

## Introduction

Nasopharyngeal carcinoma (NPC) is one of the most highly invasive and metastatic head and neck cancers in southern China and Southeast Asia (Cao et al., 2011; Torre et al., 2015). The vague symptoms and aggressiveness of NPC mean that more than 70% of patients with NPC are diagnosed with locoregionally advanced disease and often have an unfavorable prognosis (Lai et al., 2011; Chua et al., 2016). According to the National Comprehensive Cancer Network (NCCN) guidelines, platinum-based concurrent chemoradiotherapy is the standard of care for patients with locoregionally advanced nasopharyngeal carcinoma (Zhang Y. et al., 2019). However, many patients develop recurrence, distant metastasis, or both because of chemoresistance (Brabec and Kasparkova, 2005; Xie et al., 2010). Thus, the chemoresistance of NPC to platinum-based therapy not only affects treatment efficacy, but also impacts on the prognosis of patients with NPC.

Cisplatin (DDP), a DNA damaging agent, exhibits its cytotoxicity and apoptosis-inducing activities by forming DNA adducts or by targeting cancer signaling pathways (Chen et al., 2015; Amable, 2016; Wang et al., 2018). Combinations of DDP-based chemotherapies have been widely used to treat various cancers, including NPC (Agbale et al., 2016; Dugbartey et al., 2016; Sun et al., 2016). However, high-dose DDP is frequently associated with severe vomiting, ototoxicity, and hematotoxicity (Forastiere et al., 2003; Ang et al., 2014; Tang et al., 2018). In addition, clinical studies indicate that many patients acquire DDP resistance during cancer chemotherapy. Understanding the molecular mechanism underlying DDP chemoresistance is of crucial importance to develop novel therapeutic strategies to treat NPC.

In the present study, we established 5-8F and SUNE-1 DDP chemoresistance models to investigate the differential gene expression and alternative splicing (AS) events associated with NPC chemoresistance. First, we compared the chemoresistance of 5-8F DDP and 5-8F cell lines using transcriptomic sequencing and quantitative real-time reverse transcription PCR (qRT-PCR) to identify dysregulated mRNAs that participate in chemoresistance. Then, Gene Ontology (GO) and pathway analysis were performed to better understand the differentially expressed mRNAs. Next, we selected one of the most upregulated genes, *MMP1* (matrix metalloproteinase 1), to validate migration, invasion, and proliferation functions *in vitro*. Subsequently, aberrant alternative splicing events were further explored, which had not been previously reported in NPC chemoresistance. Our research provides new insights into chemoresistance of nasopharyngeal carcinoma.

## Materials and Methods

### Cell Culture

The human NPC cell lines, 5-8F, SUNE1, and HONE1; and the 5-8F DDP-chemoresistance cell line (5-8F/DDP) and SUNE1 DDP-chemoresistance cell line (SUNE1/DDP), were cultured in Roswell Park Memorial Institute (RPMI)-1640 (Gibco, Life Technologies, Carlsbad, CA, USA) supplemented with 5% fetal bovine serum. 5-8F-DDP was selected and established using increasing concentrations of DDP for more than 6 months to establish DDP-resistant cells.

### Cell Counting Kit 8 (CCK8) Assay

Cells (1 × 10^3^) were seeded in each well of 96-well plates for overnight incubation. The cells were then treated with different concentrations of DDP for 72 h. A CCK8 assay was then performed to assess cell viability, as described previously (Zhang J. et al., 2019)

### Cell Apoptosis Assays

The cell death of 5-8F, SUNE1, 5-8F/DDP, and SUNE1/DDP cell lines induced by DDP (2.5 μg/ml) was analyzed using ﬂow cytometry with Annexin-V/propidium iodide (PI) assays according to the manufacturer's instructions (BD Biosciences, Bedford, MA, USA).

### RNA Extraction and Quality Control

Total RNA was extracted from the NPC cell lines using the TRIzol reagent (Invitrogen, Grand Island, NY, USA) according to the manufacturer's instructions. RNA integrity was assessed by standard denaturing agarose gel electrophoresis. The quality and amount of RNA was assessed using a NanoDrop ND-1000 spectrophotometer (Thermo Scientiﬁc, Rockford, IL, USA) and the isolated RNAs were stored at −80°C before transcriptomic sequencing analysis.

### Construction cDNA Library

RNA (2 μg) was utilized in the RNA sample preparation, strictly according to the manufacturer's protocol. First, ribosomal RNA was removed using a RiboZero Magnetic Gold Kit (Epicentre, Madison, WI, USA), and residual RNAs were cleaned using ethanol precipitation. Sequencing libraries were generated using the rRNA-depleted RNA with KAPA Stranded RNA-Seq Library Prep Kit (Illumina, San Diego, CA, USA). RNA integrity was evaluated by using an RNA Nano 6000 Assay Kit from the Bioanalyzer 2100 system (Agilent Technologies, Santa Clara, CA, USA). The libraries were sequenced on an Illumina Hiseq 4000 platform.

### Identification of Differently Expressed Genes

The analysis of differences in mRNA expression between the two groups was performed using the DEGseq (2010) R package. The *P*-value was adjusted using the *q*-value. The threshold for significant differential expression was set as a *q*-value < 0.05 and |log2(fold change)| > 1.5 by default.

### Quantitative Real-Time Reverse Transcription PCR

qRT-PCR was performed using SYBR Green PCR Master Mix (Applied Biosystems,Foster City, CA, USA) on a CFX96 Touch Sequence Detection System (Bio-Rad, Hercules, CA, USA). All samples were normalized to internal controls and fold changes were calculated using relative quantification (2^−ΔΔ^ *^C^* ^t^). All experiments were performed in triplicate. The primer sequences are shown in **Supplementary Table S1**.

### GO and Kyoto Encyclopedia of Genes and Genomes (KEGG) Enrichment Analysis

The differentially expressed mRNAs were selected and subjected to GO and KEGG pathway analysis. For GO analysis (http://geneontology.org/), the corresponding genes were annotated and classified according to biological process (BP), cellular component (CC), and molecular function (MF). For KEGG analysis (http://www.genome.jp/kegg/), the differentially pathways were ranked by their enrichment scores.

### Alternative Splicing Detection

Replicate multivariate analysis of transcript splicing (rMATS) v4.0.2 was used to screen differential alternative splicing (AS) events across different samples (Shen et al., 2014). Various types of AS events, including alternative 3′ splice sites (A3SS, AS code: 1-, 2-), alternative 5′ splice site (A5SS, AS code: 1^, 2^), mutually exclusive exon (MXE, AS code: 0, 1-2^, 3-4^), intron retention (IR, AS code: 1^2-, 0), and exon skipping (SE, AS code: 1-2^, 0), were analyzed as previously defined (Sammeth et al., 2008). We then calculated the differential AS events with a threshold of |∆ Percent spliced in (PSI) | > 0.05 and false discovery rate (FDR) < 0.1.

### Western Blotting Analysis

Cells were lysed on ice in Radioimmunoprecipitation assay (RIPA) buffer. The protein concentration was determined using the Bradford method. Protein lysates (20 μg) were separated by sodium dodecyl sulfate polyacrylamide gel electrophoresis (SDS-PAGE) and electrophoretically transferred to a polyvinylidene fluoride membrane (Millipore, Billerica, MA, USA). The membranes were then blocked with 5% skim milk and incubated with antibodies against MMP1 (Cell Signaling Technology,Danvers, MA, USA, 1:10,000) and glyceraldehyde-3-phosphate dehydrogenase (GAPDH) (Proteintech, Rosemont, IL, USA; 1:5,000) overnight at 4°C. Species-matched secondary antibodies were then added and incubated at room temperature for 1 h. The immunoreactive proteins were detected using BeyoECLPlus (Beyotime, Shanghai, China).

### Transient Transfection and Stable Cell Line Establishment

Plasmids pEnter-MMP1-Flag and pEnter-vector were obtained from Vigene Bioscience (Rockville, MD, USA). Short interfering RNA (siRNA) oligonucleotides targeting *MMP1* were purchased from GenePharma (Shanghai China); the siRNA sequences are shown in **Supplementary Table S1**. Plasmid (2 μg) and siRNA oligonucleotide (100 nmol/L) transfections were carried out using Lipofectamine 3000 (Invitrogen) according to the manufacturer's instructions. The cells were used for further studies at 48 h after transfection.

### Wound Healing Assay

HONE1 and SUNE1 cells grown to near confluence in 6-well plates were incubated in serum-free medium for 24 h of starvation. Linear wounds were created in the cell monolayers using a P-200 pipette tip, followed by an additional 48 h of starvation. Images were captured and documented under a microscope at × 100 magnification at 0 and 24 h.

### Migration and Invasion Assays

Cells (5 × 10^4^ or 1 × 10^5^) suspended in 200 μl of serum-free medium were plated into Transwell chambers (8-μm pores, Corning, Corning, NY, USA) precoated with (migration assay) or without (invasion assay) Matrigel (BD Biosciences) and cultured at 37°C for 12 or 24 h. The cells were fixed with methyl alcohol, stained with crystal violet, and counted under a microscope in 10 random fields of view per well.

### Statistical Analysis

SPSS statistical software version 19.0 was used for data analysis (IBM Corp., Armonk, NY, USA). Chi-square and ANOVA tests were used to analyze the differences among the two groups. Statistical significance was set at a *P <* 0* *.05. The RNA sequencing data has been deposited in the Gene Expression Omnibus (accession number: GSE135083).

## Results

### Establishment of DDP-Resistant NPC Cell Lines

**Figure 1:**
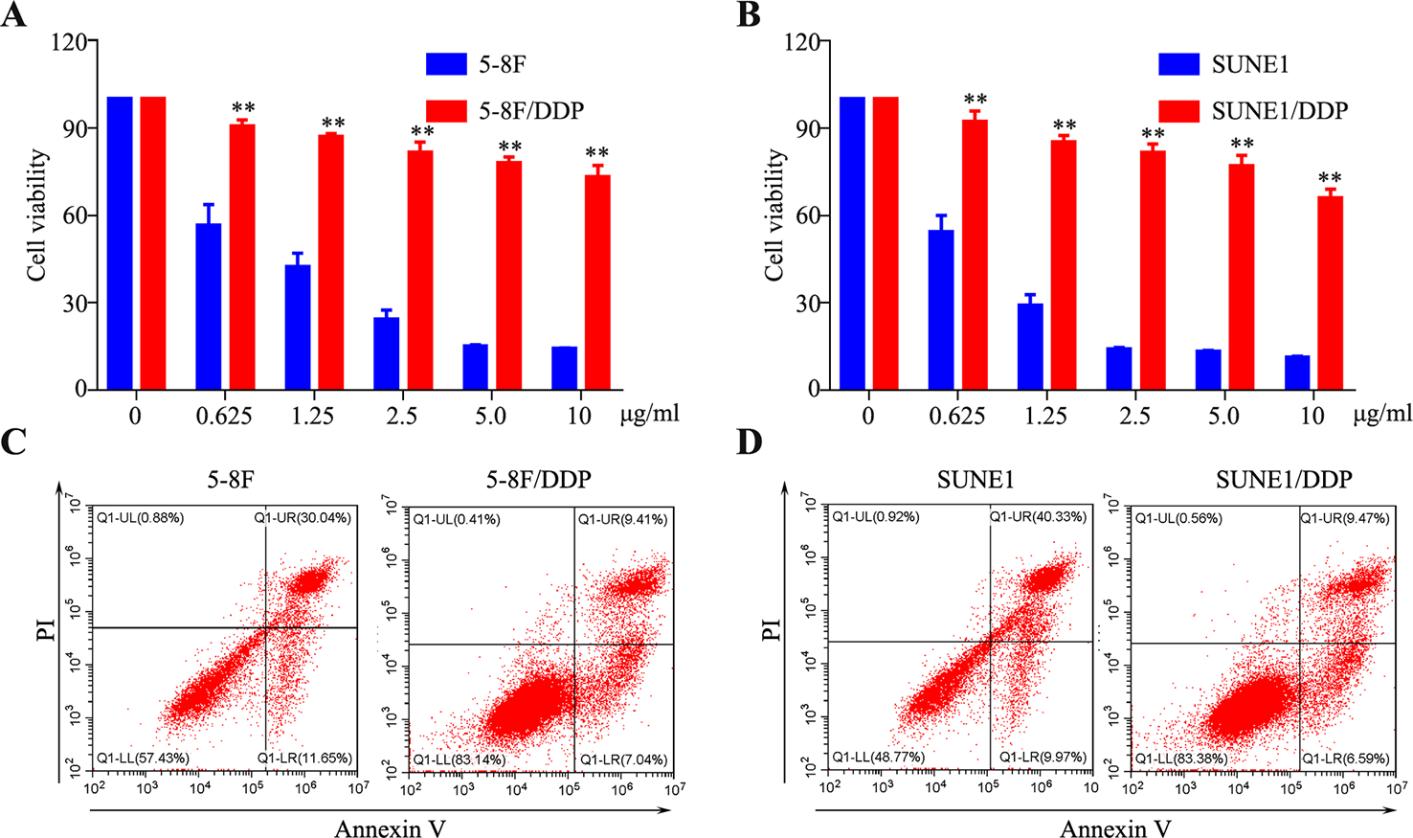
Establishment of cisplatin-resistant nasopharyngeal carcinoma (NPC) cells. **(A, B)** A CCK8 assay was conducted in parental and 5-8F/DDP **(A)** and SUNE1/DDP **(B)** cells. **(C, D)** Representative images of cell apoptosis in the 5-8F/DDP **(C)** and SUNE1/DDP **(D)** cells treated with DDP for 48 h, as determined using an Annexin-V/propidium iodide (PI) assay. All experiments were performed at least three times; data are mean ± SD. **P < 0.01 vs. control, Student's *t*-test.

To establish the DDP-resistant cells, SUNE1 and 5-8F cell lines were exposed to increasing concentrations of DDP for more than 6 months. Compared with their parental cells, chemoresistance to DDP was observed in 5-8F/DDP and SUNE1/DDP cells. As shown in **Figures 1A, B**, 2.5 μg/ml DDP led to about 50% cell growth inhibition in both SUNE1 and 5-8F cells, respectively. However, SUNE1/DDP and 5-8F/DDP cells produced resistance to the growth inhibitory properties of 2.5 μg/ml DDP. Thus, the resistant NPC cells were continuously maintained in RPMI 1600 medium containing 2.5 μg/ml DDP. To further validate the DDP-resistant NPC cell lines, flow cytometry assay was used to detect the apoptosis ability of 5-8F/DDP and SUNE1/DDP cells. As shown in **Figures 1C, D**, under 2.5 μg/ml DDP treatment, 5-8F/DDP and SUNE1/DDP cells showed a lower apoptosis rate than 5-8F and SUNE1 cells.

### RNA Sequencing of Chemoresistance Cells

**Figure 2:**
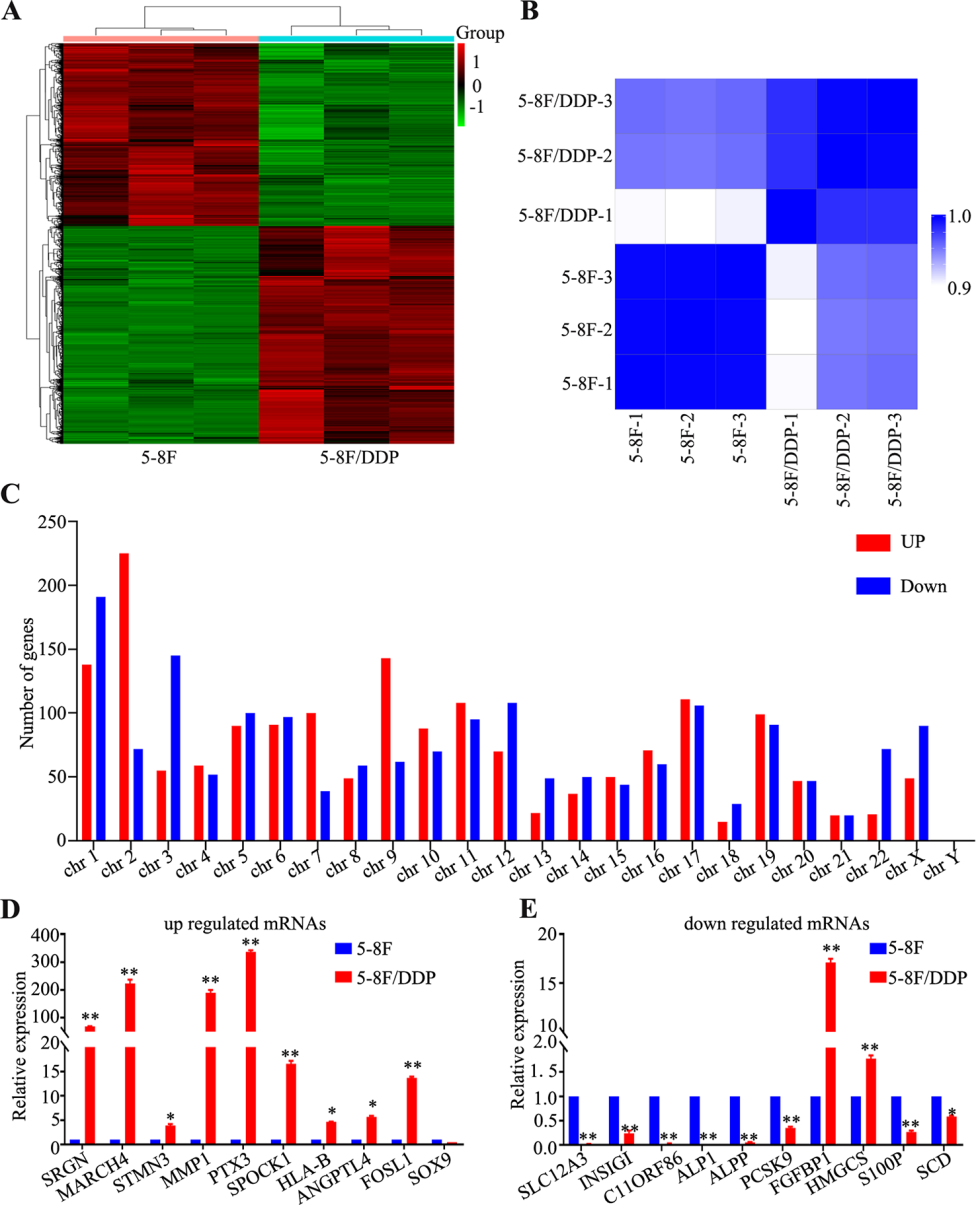
Differentially expressed genes in 5-8F/DDP cell lines compared with 5-8F cell lines. **(A)** Clustering analysis of differentially expressed genes in 5-8F and 5-8F/DDP cell lines. **(B)** Sample correlation matrix: The Spearman correlation (R^2^) was calculated and visualized by color (blue-white) in the matrix. Within the replicates for the individual sample groups, the correlation is higher than that between the sample groups. **(C)** Distribution of dysregulated mRNAs on the human chromosomes. **(D, E)** Validation of signiﬁcantly dysregulated mRNAs using qRT-PCR. The ﬁgure shows that the expression patterns of 20 mRNAs, including 10 upregulated **(D)** and 10 downregulated **(E)**, were consistent with the RNA sequencing data. All experiments were performed at least three times; data are mean ± SD. *P < 0.05, **P < 0.01 vs. control, Student's *t*-test.

5-8F and 5-8F/DDP cells were collected to perform a standard RNA sequencing analysis. The outcome of sequencing demonstrated that 11,798 mRNAs were detected in total. The mRNA expression patterns of the samples are presented as heat maps (**Figures 2A, B**). To clarify the expression signatures of the dysregulated mRNAs, we analyzed upregulated or downregulated mRNAs identiﬁed in 5-8F/DDP cell lines according to their classiﬁcation and chromosome distribution (**Figure 2C**). Compared with the 5-8F cells, 3,230 mRNAs were differentially expressed in 5-8F/DDP cells |log2(fold change)| > 1.5), among which 1,757 were upregulated and 1,473 were downregulated (**Supplementary Tables 2** and **3**). To verify the transcriptomic data, we selected the 20 most signiﬁcantly dysregulated mRNAs, including 10 upregulated mRNAs and 10 downregulated mRNAs, and then validated their expression levels using qRT-PCR. The results showed that the expression patterns of 17 mRNAs were consistent with the sequencing data (**Figures 2D, E**), which demonstrated the reliability of the RNA-seq data.

### GO and KEGG Pathway Analysis

**Figure 3:**
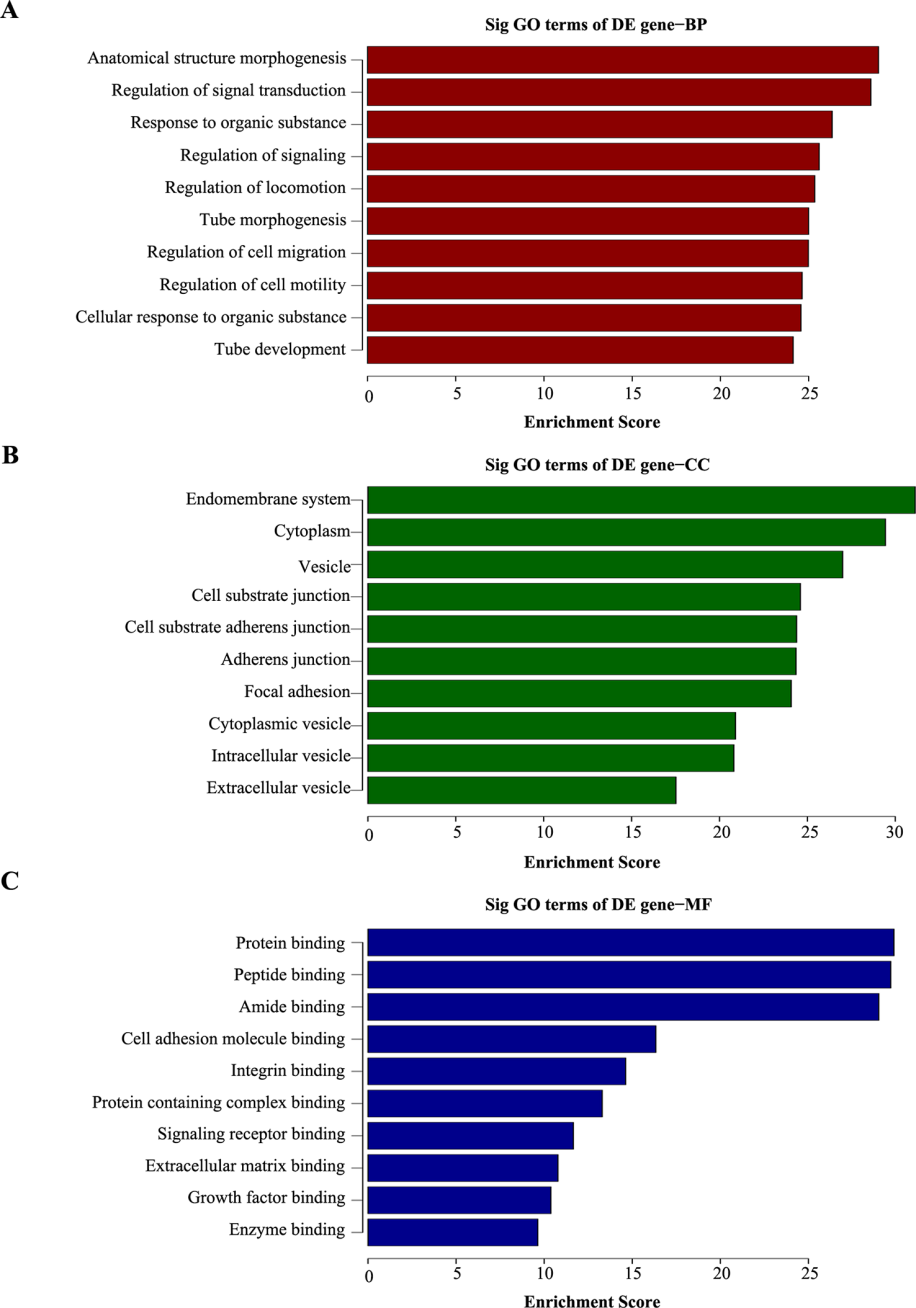
Gene ontology (GO) analysis of dysregulated genes in 5-8F/DDP cell lines compared with 5-8F cell lines. **(A–C)** The top ten enrichment score values for significantly enriched GO terms including Biological Process **(A)**, Cellular Component **(B)**, and Molecular Function **(C)**. *P*-values were calculated using the signiﬁcance test.

To explore the potential function of the differentially expressed mRNAs in chemoresistance, GO analysis was performed to describe biological process (BP), cellular component (CC) and molecular function (MF; **Supplementary Tables 4**–**6**). The GO terms were determined by calculating the Enrichment Score (*P* < 0.05). The aberrantly expressed genes were mainly enriched for GO terms related to regulation of anatomical structure morphogenesis, regulation of signal transduction, and response to organic substance involved (BP); endomembrane system, cytoplasm region, and vesicle (CC); and protein binding, peptidase binding, and amide binding activity (MF). The top ten highest and most signiﬁcant GO terms are shown in **Figures 3A–C**.

**Figure 4:**
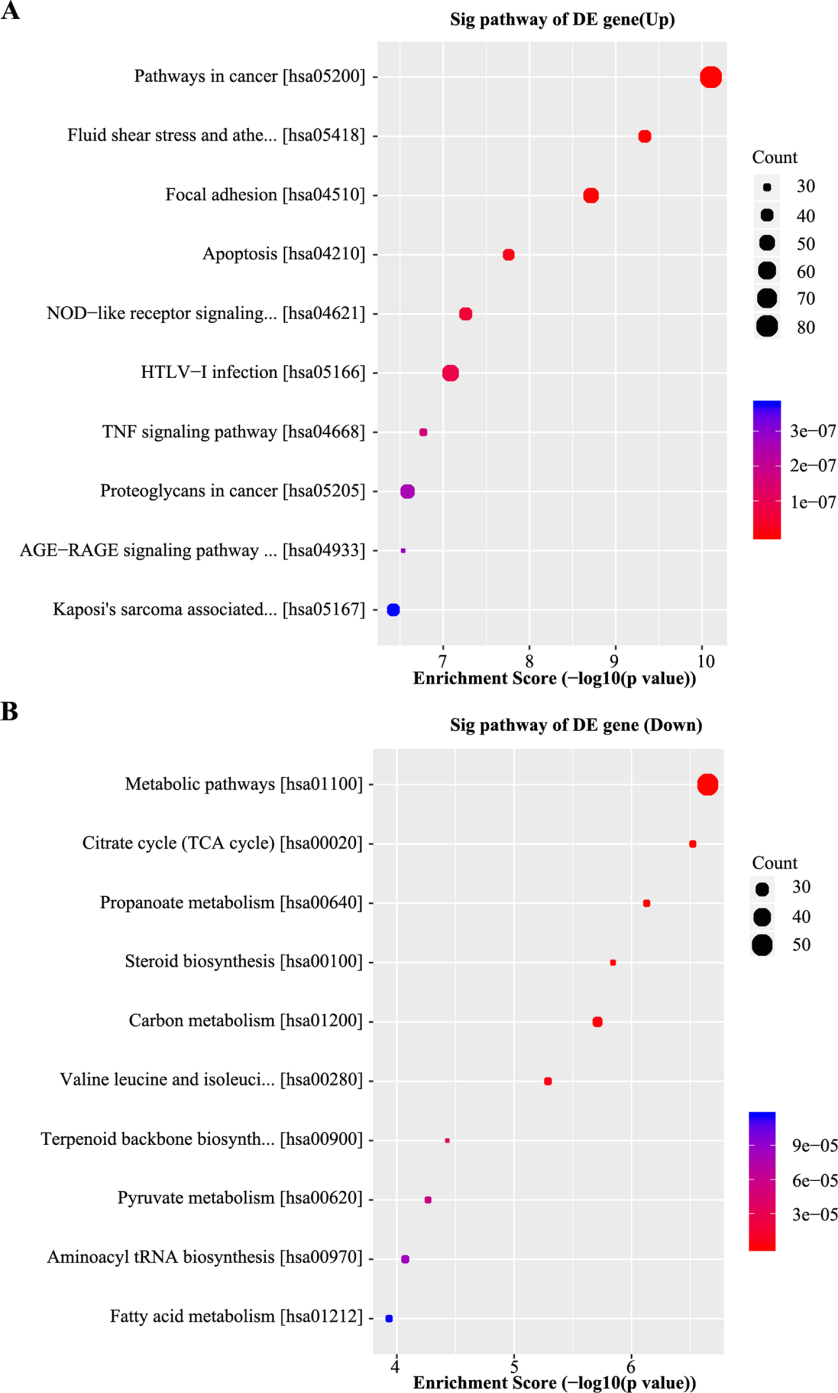
Kyoto encyclopedia of genes and genomes (KEGG) analyses of differentially expressed genes. **(A)** Scatter plot of the top 10 pathways enriched for the upregulated genes. The result indicated that these genes are involved in pathways in cancer, fluid shear stress, atherosclerosis, focal adhesion, and apoptosis. **(B)** Scatter plot of the top 10 pathways enriched for the downregulated genes. The related pathways include metabolic pathways, citrate cycle (TCA cycle), propanoate metabolism, and steroid biosynthesis. The Q-value represents the corrected *P-*value.

Pathway analysis based on the KEGG database was performed, which identiﬁed 20 pathways with signiﬁcant differences (*P* < 0.05) in gene expression (**Supplementary Table 7**). The pathway terms of top ten highest Enrichment Scores are shown in **Figures 4A, B**. The pathway analysis results suggested that the upregulated mRNAs were part of several signaling pathways, including pathways in cancer (hsa05200), fluid shear stress and atherosclerosis (hsa05418), focal adhesion (hsa04510), and the apoptosis pathway (hsa04210). However, the downregulated mRNAs participated in metabolic pathways (hsa01100), citrate cycle (TCA cycle) (hsa00020), propanoate metabolism (hsa00640), and steroid biosynthesis pathways (hsa00100), which may be associated with NPC chemoresistance.

### Alternative Splicing Analysis

**Figure 5:**
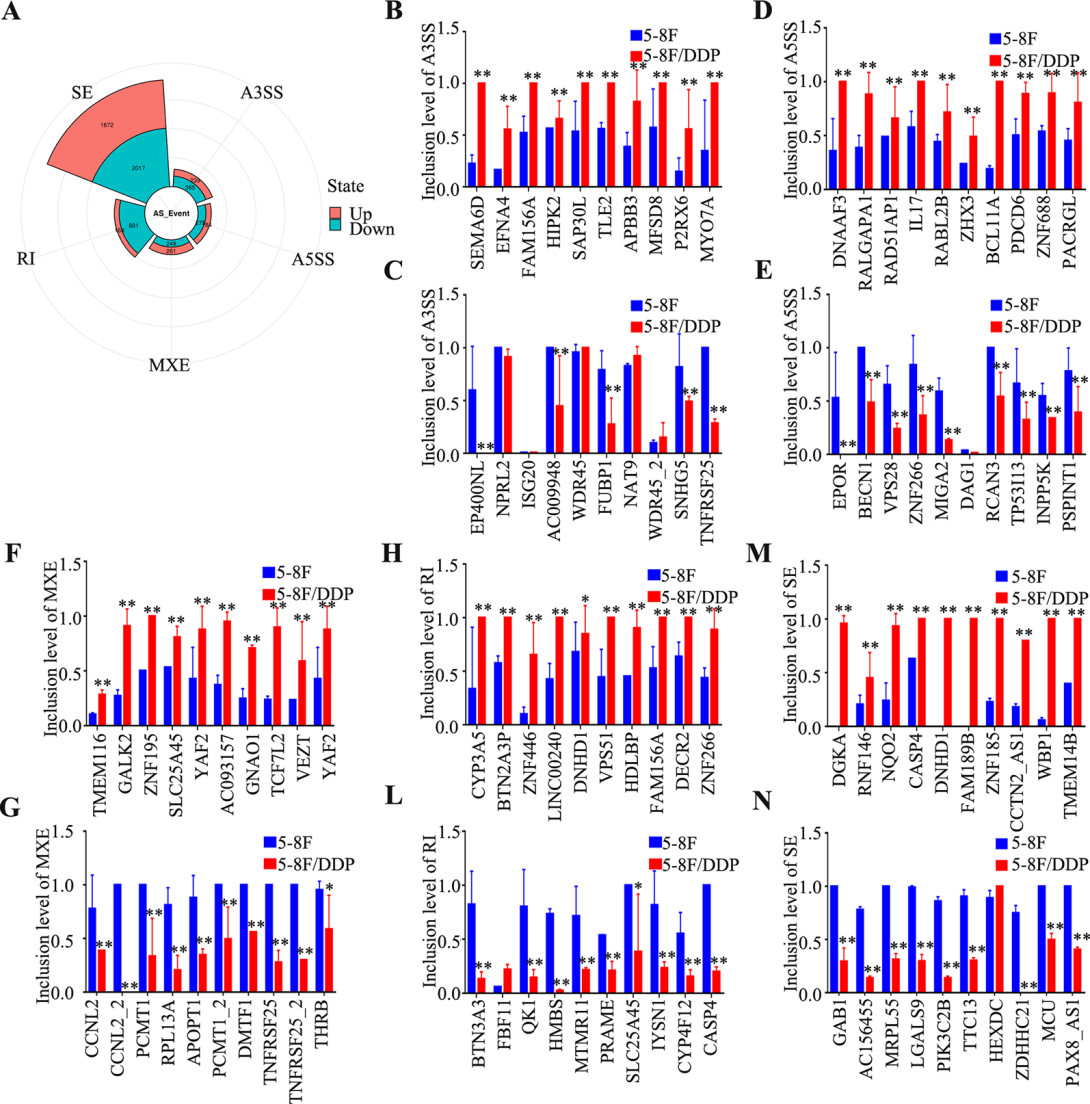
Characteristics of alternative splicing events. **(A)** Pie chart of the percentages of alternative splicing events detected in 5-8F and 5-8F/DDP cell lines. **(B–N)** The differential inclusion level of the top 10 upregulated and downregulated genes in A3SS **(B, C)**, A5SS **(D, E)**, MXE **(F, G)**, RI **(L–H)**, and SE **(M, N)**. All experiments were performed at least three times; data are mean ± SD. *P < 0.05, **P < 0.01 vs. control, Student's *t*-test.

To clarify the potential AS in 5-8F/DDP cells, A3SS, A5SS, MXE, RI, and SE events were analyzed. As shown in **Figure 5A**, 6310 AS events (2,524 upregulated and 3,786 downregulated; **Supplementary Table 8**) were identified as differentially expressed in 5-8F/DDP cells compared with 5-8F cells. The percentage of A3SS, A5SS, MXE, RI, and SE AS events were 9.6%, 7.2%, 8.2%, 16.6%, and 58.4%. Thus, SE was one of the most frequent AS events. The results showed that AS events were extensive and complicated in chemoresistance. To determine the signatures of the dysregulated AS events, the top ten upregulated or downregulated AS events in A3SS, A5SS, MXE, RI, and SE were validated using rMATS (**Figures 5B–N**). The results indicated that AS is an important molecular event and might play a vital role in chemoresistance in NPC.

### Silencing *MMP1* Promotes NPC Cell Migration and Invasion* In Vitro*

**Figure 6:**
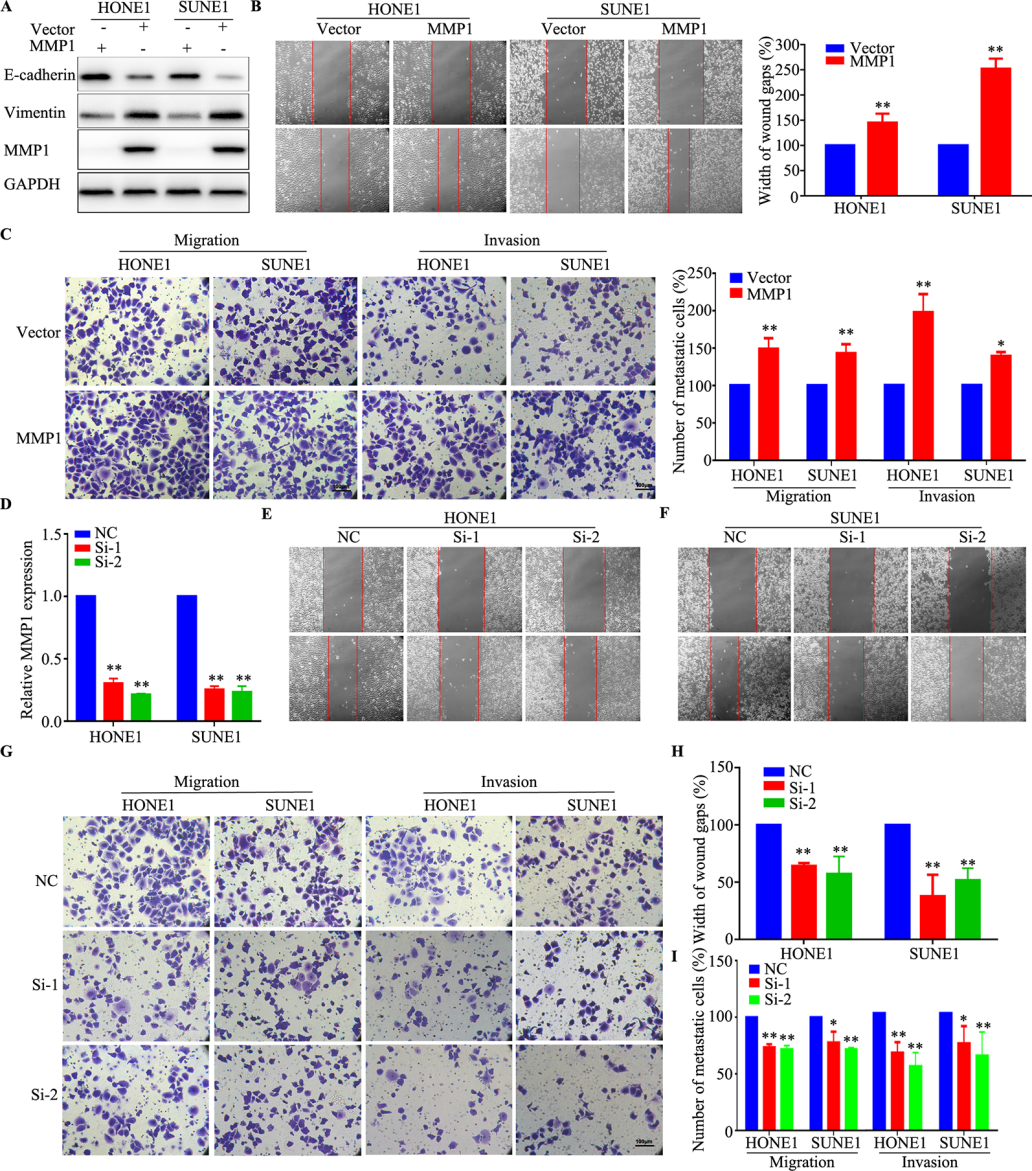
MMP1 increases the migration and invasion of NPC cells *in vitro*. **(A)** Western blotting analysis of MMP1 expression in HONE1 and SUNE1 cells overexpressing *MMP1*. **(B, C)** Overexpression of *MMP1* increased the migration **(B)** and invasion **(C)** ability of HONE1 and SUNE1 cells. **(D)** qPCR analysis of *MMP1* silencing in HONE1 and SUNE1 cells. **(E*–*I)** Silencing of *MMPI* increased the migration **(E, F, H)** and invasion **(G, I)** ability of HONE1 and SUNE1 cells. All experiments were performed at least three times; data are mean ± SD. *P < 0.05, **P < 0.01 vs. control, Student's *t-*test.

To evaluate whether aberrant expression of *MMP1*, which was one of the most upregulated mRNAs in the chemoresistant cell lines, affects the metastasis ability of NPC cells, HONE1, and SUNE1 cells were transiently transfected with *MMP1* plasmid and siRNAs targeting MMP1. As shown in **Figure 6A**, western blotting validated that MMP1 protein level was obviously elevated, meanwhile, increased vimentin expression and decreased E-cadherin expression after overexpression of MMP1 in NPC cells. The wound healing and Transwell migration and invasion assays showed that the cell migration, and invasion abilities of HONE1 and SUNE1 cells stably overexpressing MMP1 were remarkably reduced compared with that of cells transfected with the vector plasmid (**Figures 6B, C**). Conversely, silencing *MMP1* increased the migratory and invasive abilities of NPC cells (**Figures 6D–I**). These results suggest that *MMP1* inhibits NPC cell migration and invasion *in vitro*.

## Discussion

NPC is a malignancy arising from the nasopharyngeal epithelium, and it is mainly endemic in Southeast Asia and China (Tsao et al., 2014). NPC is frequently diagnosed with locoregionally advanced disease because of its anatomic location and ambiguous symptoms. Radiotherapy combined with chemotherapy is the standard therapeutic approach to treat NPC. However, because many patients develop either intrinsic or acquired drug resistance to DDP, clinical treatment failure rates remain high. The remarkable difference in treatment benefits might be associated with genetic factors. In the present study, we compared the mRNA expression differences between 5-8F/DDP and 5-8F cells, and further explored the expression and function of a dysregulated gene and AS, which might regulate chemoresistance in NPC.

Accumulating evidence demonstrates the close association between chemotherapy resistance and dysregulated gene expression. Several studies have confirmed that the aberrant expression of certain mRNAs is involved in NPC chemoresistance. Peng et al. (2019) showed that hypermethylation of *ARNTL* (encoding aryl hydrocarbon receptor nuclear translocator like) promotes NPC tumorigenesis and inhibits cisplatin sensitivity by activating *CDK5* (cyclin dependent kinase 5) transcription. Li et al. found that downregulation of *FOXM1* (forkhead box M1) could improve the sensitivity of NPC cells to cisplatin *via* inhibition of MRN (Mre11-Rad50-Nbs1)-ATM (ataxia-telangiectasia mutated) mediated DNA repair (Li D. et al., 2019). Zhang et al. found that Epstein–Barr virus (EBV) activation of ATR (ATM and Rad3-related)-mediated DNA damage response might result in chemotherapy resistance to CDDP (DDP) and 5-FU (Fluorouracil) in NPC (Zhang B. et al., 2019). However, few studies have systematically explored the relationships between dysregulated expression of mRNAs and chemoresistance in stable DDP-resistant NPC cells.

In the present study, RNA sequencing based on stable DDP-resistant cells found that 3,230 mRNAs were aberrantly regulated in NPC chemoresistance. QRT-PCR showed that serglycin (*SRGN*), *MARCH4* (membrane associated ring-CH-type finger 4), *MMP1*, *PTX3* (pentraxin 3), *SPOCK1* (SPARC (osteonectin), cwcv and kazal like domains proteoglycan 1), and *FOSL1* (FOS like 1, AP-1 transcription factor subunit) were significantly upregulated. SRGN is a proteoglycan that was first identified as secreted by hematopoietic cells. Chu et al. found that SRGN upregulated the CD44 receptor in an autocrine manner to maintain self-renewal in nasopharyngeal carcinoma cells by reciprocally activating the mitogen activated kinase (MAPK)/β-catenin axis (Chu et al., 2016). The matrix metalloprotein family (MMP), including *MMP1*, *MMP2*, *MMP14*, *MMP19*, and *MMP28*, were identified significantly upregulated genes in chemoresistant cells. Liu et al. also identified that overexpression of *MMP19* confers cisplatin sensitivity in nasopharyngeal carcinoma cells, which was consistent with our results (Liu et al., 2013). Our functional assays validated that MMP1 could increase NPC proliferation, migration, and invasion, which indicated that MMPs play an important role in NPC chemoresistance.

GO analysis predicated that the aberrant mRNAs were associated with BP, CC, and MF in NPC. The GO terms such as regulation of signaling and regulation of cell migration and motility in BP predicted that the associated gene products led to the chemoresistance of NPC. The results of pathway analysis showed that the dysregulated mRNAs were associated with 20 signaling pathways in NPC. The correlation between these pathway and tumor progression, including NPC, has been proven in previous studies. Especially, focal adhesion (Liu et al., 2018; Li Y. et al., 2019; Yuan et al., 2019; Yu et al., 2019), apoptosis (Li M. et al., 2019; Liang et al., 2019; Wang et al., 2019; Xie et al., 2019), and the tumor necrosis factor (TNF) signaling pathway (Lu et al., 2011; Ou et al., 2015; Huang et al., 2017; Deng et al., 2018) have been reported to be closely related to NPC development.

AS changes are frequently observed in cancer, and AS is starting to be recognized as an important signature in tumor progression and therapy (Climente-Gonzalez et al., 2017). However, the impact and relevance of AS on tumorigenesis and chemoresistance remain mostly unknown. In the present study, we carried out a systematic analysis to characterize the AS events and found that AS (A3SS, A5SS, MXE, RI, SE) were frequently dysregulated in DDP chemoresistant cells, especially RI (13.7%). Recently, several studies confirmed that AS is also involved in tumor malignant features. Downregulation of *QK1* (QKI, KH domain containing RNA binding) led to the alternative splicing change in *NUMB* (NUMB endocytic adaptor protein), which promoted cell proliferation (Zong et al., 2014). An SE event in *MST1R* (macrophage stimulating 1 receptor) has been related to the acquisition of cell motility during cancer cell invasion (Ghigna et al., 2005). The small-molecule modulators of pre-mRNA splicing are capable of restoring the original *BRAF* (B-raf proto-oncogene, serine/threonine kinase) splicing and rescue growth of therapy-resistant cells (Salton et al., 2015). The modulation of these events can recapitulate the tumor phenotype or revert to a normal phenotype (Ghigna et al., 2010; Bechara et al., 2013). Thus, determining alterations in AS is essential to understand the functional chemoresistance in cancer and targeting AS might provide a new way to alleviate chemoresistance.

In conclusion, dysregulated gene- and AS-mediated DDP chemoresistance might play important roles in NPC development and progression. These findings indicated that targeting aberrantly expressed genes and AS might provide novel insights into the pathogenesis of NPC and contribute to the development of new therapeutic strategies to treat NPC.

## Data Availability Statement

All datasets generated and analyzed for this study have been deposited in the Gene Expression Omnibus (accession number: GSE135083).

## Author Contributions

JZha, HJ, and TX designed the research. TX, JZhe, YT, RL, BW, JL, and AX acquired and analyzed the data. JZha, HJ, and YY wrote the manuscript.

## Funding

This work was supported by grants from the Social Science and Technology Development Key Project of Dongguan (201750715046462); Guangzhou Key Medical Discipline Construction Project Fund (B195002004042); and Open Funds of State Key Laboratory of Oncology in South China (KY013711).

## Conflict of Interest

The authors declare that the research was conducted in the absence of any commercial or financial relationships that could be construed as a potential conflict of interest.

## References

[B1] Agbale C. M. Cardoso M. H. Galyuon I. K. Franco O. L. (2016). Designing metallodrugs with nuclease and protease activity. Metallomics 8, 1159–1169. 10.1039/C6MT00133E 27714031

[B2] Amable L. (2016). Cisplatin resistance and opportunities for precision medicine. Pharmacol. Res. 106, 27–36. 10.1016/j.phrs.2016.01.001 26804248

[B3] Ang K. K. Zhang Q. Rosenthal D. I. Nguyen-Tan P. F. Sherman E. J. Weber R. S. (2014). Randomized phase III trial of concurrent accelerated radiation plus cisplatin with or without cetuximab for stage III to IV head and neck carcinoma. RTOG 0522. J. Clin. Oncol. 32, 2940–2950. 10.1200/JCO.2013.53.5633 25154822 PMC4162493

[B4] Bechara E. G. Sebestyen E. Bernardis I. Eyras E. Valcarcel J. (2013). RBM5, 6, and 10 differentially regulate NUMB alternative splicing to control cancer cell proliferation. Mol. Cell 52, 720–733. 10.1016/j.molcel.2013.11.010 24332178

[B5] Brabec V. Kasparkova J. (2005). Modifications of DNA by platinum complexes. Relation to resistance of tumors to platinum antitumor drugs. Drug Resist. Updates. 8, 131–146. 10.1016/j.drup.2005.04.006 15894512

[B6] Cao S. M. Simons M. J. Qian C. N. (2011). The prevalence and prevention of nasopharyngeal carcinoma in China. Chin J. Cancer 30, 114–119. 10.5732/cjc.010.10377 21272443 PMC4013340

[B7] Chen S. H. Kuo C. C. Li C. F. Cheung C. H. Tsou T. C. Chiang H. C. (2015). O(6)-methylguanine DNA methyltransferase repairs platinum-DNA adducts following cisplatin treatment and predicts prognoses of nasopharyngeal carcinoma. Int. J. Cancer. 137, 1291–1305. 10.1002/ijc.29486 25693518

[B8] Chu Q. Huang H. Huang T. Cao L. Peng L. Shi S. (2016). Extracellular serglycin upregulates the CD44 receptor in an autocrine manner to maintain self-renewal in nasopharyngeal carcinoma cells by reciprocally activating the MAPK/β-catenin axis. Cell Death Dis. 7, e2456. 10.1038/cddis.2016.287 27809309 PMC5260886

[B9] Chua M. L. K. Wee J. T. S. Hui E. P. Chan A. T. C. (2016). Nasopharyngeal carcinoma. Lancet 387, 1012–1024. 10.1016/S0140-6736(15)00055-0 26321262

[B10] Climente-Gonzalez H. Porta-Pardo E. Godzik A. Eyras E. (2017). The functional impact of alternative splicing in cancer. Cell Rep. 20, 2215–2226. 10.1016/j.celrep.2017.08.012 28854369

[B11] Deng C. Lin Y. X. Qi X. K. He G. P. Zhang Y. Zhang H. J. (2018). TNFRSF19 Inhibits TGFbeta Signaling through Interaction with TGFbeta Receptor Type I to Promote Tumorigenesis. Cancer Res. 78, 3469–3483. 10.1158/0008-5472.CAN-17-3205 29735548

[B12] Dugbartey G. J. Peppone L. J. de, Graaf I. A. (2016). An integrative view of cisplatin-induced renal and cardiac toxicities: molecular mechanisms, current treatment challenges and potential protective measures. Toxicology 371, 58–66. 10.1016/j.tox.2016.10.001 27717837 PMC5586594

[B13] Forastiere A. A. Goepfert H. Maor M. Pajak T. F. Weber R. Morrison W. (2003). Concurrent chemotherapy and radiotherapy for organ preservation in advanced laryngeal cancer. N Engl. J. Med. 349, 2091–2098. 10.1056/NEJMoa031317 14645636

[B14] Ghigna C. Giordano S. Shen H. Benvenuto F. Castiglioni F. Comoglio P. M. (2005). Cell motility is controlled by SF2/ASF through alternative splicing of the Ron protooncogene. Mol. Cell 20, 881–890. 10.1016/j.molcel.2005.10.026 16364913

[B15] Ghigna C. De, Toledo M. Bonomi S. Valacca C. Gallo S. Apicella M. (2010). Pro-metastatic splicing of Ron proto-oncogene mRNA can be reversed: therapeutic potential of bifunctional oligonucleotides and indole derivatives. RNA Biol. 7, 495–503. 10.4161/rna.7.4.12744 20864806

[B16] Huang T. Yin L. Wu J. Gu J. J. Ding K. Zhang N. (2017). TNFAIP3 inhibits migration and invasion in nasopharyngeal carcinoma by suppressing epithelial mesenchymal transition. Neoplasma 64, 389–394. 10.4149/neo_2017_309 28253718

[B17] Lai S. Z. Li W. F. Chen L. Luo W. Chen Y. Y. Liu L. Z. (2011). How does intensity-modulated radiotherapy versus conventional two-dimensional radiotherapy influence the treatment results in nasopharyngeal carcinoma patients? Int. J. Radiat. Oncol. 80, 661–668. 10.1016/j.ijrobp.2010.03.024 20643517

[B18] Li W. Zhou X. Ye J. Jia Q. (2013). Development of a γ-alumina- nanoparticle-functionalized porous polymer monolith for the enrichment of Sudan dyes in red wine samples. J. Sep Sci. 36, 3330–3307. 10.1002/jssc.201300754 23956065

[B19] Li D. Ye L. Lei Y. Wan J. Chen H. (2019). Downregulation of FoxM1 sensitizes nasopharyngeal carcinoma cells to cisplatin *via* inhibition of MRN-ATM-mediated DNA repair. BMB Rep. 52, 208–213. 10.5483/BMBRep.2019.52.3.249 30638177 PMC6476488

[B20] Li M. Liu Y. Wei Y. Wu C. Meng H. Niu W. (2019). Zinc-finger protein YY1 suppresses tumor growth of human nasopharyngeal carcinoma by inactivating c-Myc-mediated microRNA-141 transcription. J. Biol. Chem. 294, 6172–6187. 10.1074/jbc.RA118.006281 30718276 PMC6463721

[B21] Li Y. He Q. Wen X. Hong X. Yang X. Tang X. (2019). EZH2-DNMT1-mediated epigenetic silencing of miR-142-3p promotes metastasis through targeting ZEB2 in nasopharyngeal carcinoma. Cell Death Differ 26, 1089–1106. 10.1038/s41418-018-0208-2 30353102 PMC6748116

[B22] Liang T. S. Zheng Y. J. Wang J. Zhao J. Y. Yang D. K. Liu Z. S. (2019). MicroRNA-506 inhibits tumor growth and metastasis in nasopharyngeal carcinoma through the inactivation of the Wnt/beta-catenin signaling pathway by down-regulating LHX2. J. Exp. Clin. Cancer Res. 38, 97. 10.1186/s13046-019-1023-4 30791932 PMC6385449

[B23] Liu R. Y. Dong Z. Liu J. Zhou L. Huang W. Khoo S. K. (2013). Overexpression of asparagine synthetase and matrix metalloproteinase 19 confers cisplatin sensitivity in nasopharyngeal carcinoma cells. Mol. Cancer Ther. 12, 2157–2166. 10.1158/1535-7163.MCT-12-1190 23956056 PMC3795908

[B24] Liu S. C. Hsu T. Chang Y. S. Chung A. K. Jiang S. S. OuYang C. N. (2018). Cytoplasmic LIF reprograms invasive mode to enhance NPC dissemination through modulating YAP1-FAK/PXN signaling. Nat. Commun. 9, 5105. 10.1038/s41467-018-07660-6 30504771 PMC6269507

[B25] Lu X. Qian C. N. Mu Y. G. Li N. W. Li S. Zhang H. B. (2011). Serum CCL2 and serum TNF-alpha–two new biomarkers predict bone invasion, post-treatment distant metastasis and poor overall survival in nasopharyngeal carcinoma. Eur. J. Cancer. 47, 339–346. 10.1016/j.ejca.2010.09.025 20951575

[B26] Ou C. Sun Z. Zhang H. Xiong W. Ma J. Zhou M. (2015). SPLUNC1 reduces the inflammatory response of nasopharyngeal carcinoma cells infected with the EB virus by inhibiting the TLR9/NF-kappaB pathway. Oncol. Rep. 33, 2779–2788. 10.3892/or.2015.3913 25891128

[B27] Peng H. Zhang J. Zhang P. P. Chen L. Tang L. L. Yang X. J. (2019). ARNTL hypermethylation promotes tumorigenesis and inhibits cisplatin sensitivity by activating CDK5 transcription in nasopharyngeal carcinoma. J. Exp. Clin. Cancer Res. 38 (1), 11. 10.1186/s13046-018-0997-7 30621723 PMC6325889

[B28] Salton M. Kasprzak W. K. Voss T. Shapiro B. A. Poulikakos P. I. Misteli T. (2015). Inhibition of vemurafenib-resistant melanoma by interference with pre-mRNA splicing. Nat. Commun. 6, 7103. 10.1038/ncomms8103 25971842 PMC4435825

[B29] Sammeth M. Foissac S. Guigó R. (2008). A general definition and nomenclature for alternative splicing events. PloS Comput. Biol. 4 (8), e1000147. 10.1371/journal.pcbi.1000147 18688268 PMC2467475

[B30] Shen S. Park J. W. Lu Z. X. Lin L. Henry M. D. Wu Y. N. (2014). rMATS: robust and flexible detection of differential alternative splicing from replicate RNA-Seq data. Proc. Natl. Acad. Sci. U S A. 111, E5593–E5601. 10.1073/pnas 25480548 PMC4280593

[B31] Sun Y. Li W. F. Chen N. Y. Zhang N. Hu G. Q. Xie F. Y. (2016). Induction chemotherapy plus concurrent chemoradiotherapy versus concurrent chemoradiotherapy alone in locoregionally advanced nasopharyngeal carcinoma: a phase 3, multicentre, randomised controlled trial. Lancet Oncol. 17, 1509–1520. 10.1016/S1470-2045(16)30410-7 27686945

[B32] Tang L. Q. Chen D. P. Guo L. Mo H. Y. Huang Y. Guo S. S. (2018). Concurrent chemoradiotherapy with nedaplatin versus cisplatin in stage II-IVB nasopharyngeal carcinoma: an open-label, non-inferiority, randomised phase 3 trial. Lancet Oncol. 19, 461–473. 10.1016/S1470-2045(18)30104-9 29501366

[B33] Torre L. A. Bray F. Siegel R. L. Ferlay J. Lortet-Tieulent J. Jemal A. (2015). Global cancer statistics 2012. CA-Cancer J. Clin. 65, 87–108. 10.3322/caac.21262 25651787

[B34] Tsao S. W. Yip Y. L. Tsang C. M. Pang P. S. Lau V. M. Zhang G. (2014). Etiological factors of nasopharyngeal carcinoma. Oncol. 50, 330–338. 10.1016/j.oraloncology.2014.02.006 24630258

[B35] Wang K. M. Chen Z. M. Long L. M. Tao Y. M. Wu Q. M. Xiang M. M. (2018). iTRAQ-based quantitative proteomic analysis of differentially expressed proteins in chemoresistant nasopharyngeal carcinoma. Cancer Biol. Ther. 19, 809–824. 10.1080/15384047.2018.1472192 30067426 PMC6154836

[B36] Wang D. Luo H. Huo Z. Chen M. Han Z. Hung M. (2019). Irradiation-induced dynamic changes of gene signatures reveal gain of metastatic ability in nasopharyngeal carcinoma. Am. J. Cancer Res. 9, 479–495. 30949405 PMC6448059

[B37] Xie S. M. Fang W. Y. Liu T. F. Yao K. T. Zhong X. Y. (2010). Association of ABCC2 and CDDP-resistance in two sublines resistant to CDDP derived from a human nasopharyngeal carcinoma cell line. J. Oncol. 2010, 915046. 10.1155/2010/915046 20628484 PMC2902222

[B38] Xie X. Lin W. Zheng W. Chen T. Yang H. Sun L. (2019). Downregulation of G2/mitotic-specific cyclinB1 triggers autophagy *via* AMPK-ULK1-dependent signal pathway in nasopharyngeal carcinoma cells. Cell Death Dis. 10, 94. 10.1038/s41419-019-1369-8 30700698 PMC6353984

[B39] Yu X. Liu Y. Yin L. Peng Y. Peng Y. Gao Y. (2019). Radiation-promoted CDC6 protein stability contributes to radioresistance by regulating senescence and epithelial to mesenchymal transition. Oncogene 38, 549–563. 10.1038/s41388-018-0460-4 30158672 PMC6345673

[B40] Yuan J. Chen L. Xiao J. Qi X. K. Zhang J. Li X. (2019). SHROOM2 inhibits tumor metastasis through RhoA-ROCK pathway-dependent and -independent mechanisms in nasopharyngeal carcinoma. Cell Death Dis. 10, 58. 10.1038/s41419-019-1325-7 30683844 PMC6347642

[B41] Zhang B. Cui B. Du J. Shen X. Wang K. Chen J. (2019). ATR activated by EB virus facilitates chemotherapy resistance to cisplatin or 5-fluorouracil in human nasopharyngeal carcinoma. Cancer Manag Rese. 11, 573–585. 10.2147/CMAR.S187099 PMC6331066 30666155

[B42] Zhang J. Zheng Z. Q. Yuan Y. W. Zhang P. P. Li Y. Q. Wang Y. Q. (2019). NFAT1 hypermethylation promotes epithelial-mesenchymal transition and metastasis in nasopharyngeal carcinoma by activating ITGA6 transcription. Neoplasia 21, 311–321. 10.1016/j.neo.2019.01.006 30772768 PMC6378632

[B43] Zhang Y. Chen L. Hu G. Q. Zhang N. Zhu X. D. Yang K. Y. (2019). Gemcitabine and cisplatin induction chemotherapy in nasopharyngeal carcinoma. N Engl. J. Med. 384, 1124–1135. 10.1056/NEJMoa1905287 31150573

[B44] Zong F. Y. Fu X. Wei W. J. Luo Y. G. Heiner M. Cao L. J. (2014). The RNA-binding protein QKI suppresses cancer-associated aberrant splicing. PLoS Genet. 10, e1004289. 10.1371/journal.pgen.1004289 24722255 PMC3983035

