# Supplementary material for: Differential Expression and Alternative Splicing of Transcripts Associated With Cisplatin-Induced Chemoresistance in Nasopharyngeal Carcinoma
Source: Front Genet. 2020 Feb 25;11:52. doi: 10.3389/fgene.2020.00052 (PMC7052373; doi:10.3389/fgene.2020.00052)
Supplement: Supplementary file 1 [file Table_1.docx]

**Supplementary Table S1**. Primers used in this study

| Gene | Sequence (5’ to 3’) |
| --- | --- |
| **Real time RT-PCR primers**  *SRGN*-F | AGGTTATCCTACGCGGAGAG |
| *SRGN*-R | GTCTTTGGAAAAAGGTCAGTCCT |
| *MARCH4*-F | CTGTAAGGAGAAGACCGAGGA |
| *MARCH4*-R | ATCCACTTGATGAGGCAAGGC |
| *STMN3*-F | CCAGCACCATTTCCGCCTA |
| *STMN3*-R  *MMP1*-F  *MMP1*-R  PTX3-F  PTX3-R  SPOCK1-F  SPOCK1-R  HLA-B-F  HLA-B-R  ANGPTL4-F  ANGPTL4-R  FOSL1-F  FOSL1-R  SOX9-F  SOX9-R  SLC12A3-F  SLC12A3-R  INSIGI-F  INSIGI-R  CO11ORF86-F  CO11ORF86-R  ALP1-F  ALP1-R  ALPP-F  ALPP-R  PCSK9-F  PCSK9-R  FGFBP1-F  FGFBP1-R  HMGCS-F  HMGCS-R  S100P-F  S100P-R  SCD-F  SCD-R | TGCGGCTGTGTGTAGAAGC  CTGAAGAATGATGGGAGGCA  TGGCAAATCTGGCGTGTAA  CATCTCCTTGCGATTCTGTTTTG  CCATTCCGAGTGCTCCTGA  CCCAACCACGGCAATTTCCTA  ATCGTCTCGAAAGCGGTTCC  CAGTTCGTGAGGTTCGACAG  CAGCCGTACATGCTCTGGA  GTCCACCGACCTCCCGTTA  CCTCATGGTCTAGGTGCTTGT  CAGGCGGAGACTGACAAACTG  TCCTTCCGGGATTTTGCAGAT  AGGAAGCTCGCGGACCAGTAC  GGTGGTCCTTCTTGTGCTGCAC  CTCCACCAATGGCAAGGTCAA  GGATGTCGTTAATGGGGTCCA  TAACCACGCCAGTGCTAAAT  AAGGGAGCCAAGAACGAATA  CTGCGAAGTCAGTCCTTGC  CCACCTTCTTCTTACCTGTTGGT  TGAGGGTGTGGCTTACCAG  GATGGACGTGTAGGCTTTGCT  TACCTGTGCGGGGTCAAG  GCTGCGTAGCGATGTCCTG  AGACCCACCTCTCGCAGTC  GGAGTCCTCCTCGATGTAGTC  CTTCACAGCAAAGTGGTCTCA  GACACAGGAAAATTCATGGTCCA  GATGTGGGAATTGTTGCCCTT  ATTGTCTCTGTTCCAACTTCCAG  AAGGATGCCGTGGATAAATTGC  ACACGATGAACTCACTGAAGTC  TGTCTATGAATGGGCTCGTG  TACCTCCTCTGGAACATCACC |
| *GAPDH*-F | TGATGACATCAAGAAGGTGG |
| *GAPDH*-R  **siRNA sequences**  siRNA-NC-F  siRNA-NC-R  siRNA-*MMP1*-1-F  siRNA-*MMP1*-1-R  siRNA-*MMP1*-2-F  siRNA-*MMP1*-2-R | TTGTCATACCAGGAAATGAGC  UUCUCCGAACGUGUCACGUTT  ACGUGACACGUUCGGAGAATT  GUCAAGCAGACAUCAUGAUTT  AUCAUGAUGUCUGCUUGACTT  CUCUCCCAUUCUACUGAUATT  UAUCAGUAGAAUGGGAGAGTT |
